# Adult neurogenesis modifies excitability of the dentate gyrus

**DOI:** 10.3389/fncir.2013.00204

**Published:** 2013-12-26

**Authors:** Taruna Ikrar, Nannan Guo, Kaiwen He, Antoine Besnard, Sally Levinson, Alexis Hill, Hey-Kyoung Lee, Rene Hen, Xiangmin Xu, Amar Sahay

**Affiliations:** ^1^Department of Anatomy and Neurobiology, School of Medicine, University of CaliforniaIrvine, CA, USA; ^2^Center for Regenerative Medicine, Massachusetts General Hospital, Harvard Medical SchoolBoston, MA, USA; ^3^Department of Biology, University of MarylandCollege Park, MD, USA; ^4^The Solomon H. Snyder Department of Neuroscience, The Zanvyl-Krieger Mind/Brain Institute, Johns Hopkins UniversityBaltimore, MD, USA; ^5^Division of Integrative Neuroscience, Departments of Neuroscience and Psychiatry, Department of Pharmacology, Columbia UniversityNew York, NY, USA; ^6^Department of Biomedical Engineering, University of CaliforniaIrvine, CA, USA; ^7^Harvard Stem Cell Institute, Harvard UniversityBoston, MA, USA; ^8^Department of Psychiatry, Massachusetts General Hospital, Harvard Medical SchoolBoston, MA, USA

**Keywords:** adult neurogenesis, dentate gyrus, pattern separation, dentate granule neurons, encoding, inhibition, excitability

## Abstract

Adult-born dentate granule neurons contribute to memory encoding functions of the dentate gyrus (DG) such as pattern separation. However, local circuit-mechanisms by which adult-born neurons partake in this process are poorly understood. Computational, neuroanatomical and electrophysiological studies suggest that sparseness of activation in the granule cell layer (GCL) is conducive for pattern separation. A sparse coding scheme is thought to facilitate the distribution of similar entorhinal inputs across the GCL to decorrelate overlapping representations and minimize interference. Here we used fast voltage-sensitive dye (VSD) imaging combined with laser photostimulation and electrical stimulation to examine how selectively increasing adult DG neurogenesis influences local circuit activity and excitability. We show that DG of mice with more adult-born neurons exhibits decreased strength of neuronal activation and more restricted excitation spread in GCL while maintaining effective output to CA3c. Conversely, blockade of adult hippocampal neurogenesis changed excitability of the DG in the opposite direction. Analysis of GABAergic inhibition onto mature dentate granule neurons in the DG of mice with more adult-born neurons shows a modest readjustment of perisomatic inhibitory synaptic gain without changes in overall inhibitory tone, presynaptic properties or GABAergic innervation pattern. Retroviral labeling of connectivity in mice with more adult-born neurons showed increased number of excitatory synaptic contacts of adult-born neurons onto hilar interneurons. Together, these studies demonstrate that adult hippocampal neurogenesis modifies excitability of mature dentate granule neurons and that this non-cell autonomous effect may be mediated by local circuit mechanisms such as excitatory drive onto hilar interneurons. Modulation of DG excitability by adult-born dentate granule neurons may enhance sparse coding in the GCL to influence pattern separation.

## Introduction

The dentate gyrus (DG) is thought to support pattern separation, a process by which similar inputs are transformed into more distinct outputs (Treves and Rolls, 1992; McClelland and Goddard, 1996; Gilbert et al., 2001; Rolls and Kesner, 2006; Leutgeb et al., 2007; McHugh et al., 2007; Treves et al., 2008; Yassa and Stark, 2011). Sparseness of encoding in DG may facilitate pattern separation by enhancing global remapping, a process by which similar representations are encoded by non-overlapping neuronal ensembles. A sparse coding mechanism in DG coupled with sparse functional connectivity between DG and CA3 may ensure that distinct ensembles of dentate granule neurons recruit non-overlapping CA3 cell assemblies for memory retrieval (O'Reilly and McClelland, 1994; Rolls, 1996).

The adult DG is host to ongoing neurogenesis and is transiently populated by a reservoir of excitable young adult-born dentate granule neurons (Ming and Song, 2011) that matures into a larger, stable population of relatively silent neurons with high input specificity (Jung and McNaughton, 1993; Chawla et al., 2005; Aimone et al., 2011; Dieni et al., 2012; Marin-Burgin et al., 2012; Neunuebel and Knierim, 2012). Recent studies have shown that adult-born neurons are involved in pattern separation (Clelland et al., 2009; Tronel et al., 2010; Sahay et al., 2011b; Nakashiba et al., 2012; Niibori et al., 2012; Kheirbek et al., 2012a). Furthermore, mature dentate granule neurons preferentially partake in global remapping (Neunuebel and Knierim, 2012; Deng et al., 2013) in the DG supporting a role for this population as a cellular substrate for pattern separation. These observations beg the questions how adult-born neurons contribute to pattern separation in the DG (Piatti et al., 2013) and whether they act as encoding units and in addition, as modulators of activity of mature neurons to dictate sparse encoding in DG.

To determine the impact of adult hippocampal neurogenesis on sparseness of activation in DG, we asked whether increasing or ablating adult hippocampal neurogenesis changes the excitability of the DG, the vast majority of which is comprised of mature dentate granule neurons. We used fast VSDI combined with laser photostimulation and electrical stimulation of the granule cell layer (GCL) to visualize local circuit activity and signal propagation in DG-CA3 circuitry of mice in which we genetically increased the number of adult-born dentate granule neurons (referred to as *iBax^nestin^* mice) (Sahay et al., 2011b). We found a reduction in spread of activity and strength of neuronal activation in the DG of *iBax^nestin^* mice relative to controls. Interestingly, DG to CA3c output in response to stimulation remained unchanged. Conversely, mice in which adult hippocampal neurogenesis was blocked by targeted x-irradiation showed an opposite trend in change in DG excitability. Although increasing adult hippocampal neurogenesis did not affect GABAergic innervation of the DG, recordings of perisomatic miniature inhibitory post-synaptic currents (mIPSCs) in mature dentate granule neurons showed a redistribution of the gain of inhibitory post-synaptic strength without a change in average inhibitory strength or presynaptic properties. In addition, retroviral ablation of *Bax* in neural progenitors increased survival of adult-born neurons and resulted in a proportionate increase in excitatory contacts with hilar interneurons. This provides direct evidence for how increasing adult hippocampal neurogenesis increases excitatory drive onto hilar interneurons. Together, these results demonstrate that levels of adult hippocampal neurogenesis modify DG excitability and that this non-cell autonomous effect of adult-born neurons may be mediated by recruitment of local hilar inhibitory circuitry. Such a modulatory role for adult-born neurons on excitability of the DG may facilitate pattern separation by global remapping by affecting both input specificity of mature neurons as well as sparseness of activation in the DG (Lacefield et al., 2010; Ming and Song, 2011; Sahay et al., 2011a).

## Materials and methods

### Mouse lines and animal care

*Nestin CreER*^*T*2^*; Bax ^f/f^* (NCff) mice were generated by interbreeding *Nestin CreER*^*T*2^*; Bax ^f/f^*and *Bax ^f/f^* mice as previously described (Sahay et al., 2011b). *Bax ^f/f^* mice and *Bax* ^+/+^ mice were generated from interbreeding *Bax ^f/+^* mice. To induce CreER^*T*2^ mediated recombination of *Bax* in neural stem cells in the adult brain, mice were given 2 mg of tamoxifen (TAM), once a day, intraperitoneally for 5 consecutive days. Ten mg/ml TAM (Sigma, T-5648) solution was prepared in corn oil containing 10 percent ethanol. For vehicle (Veh), an identical volume of corn oil with 10 percent ethanol was injected intraperitoneally, once a day for 5 consecutive days. Mice were housed four to five per cage in a 12 h (06:00–18:00) light-dark colony room at 22°C and had free access to food and water. All animals were handled and experiments were conducted in accordance with procedures approved by the Institutional Animal Care and Use Committee at the University of California at Irvine, University of Maryland, Columbia University, the New York State Psychiatric Institute and Massachusetts General Hospital subcommittee on Research Animal Care in accordance with NIH guidelines.

### Focal x-irradiation of hippocampus

Eight–nine weeks old C57BL/6J male mice were anesthetized with sodium pentobarbital (administered intraperitoneally at 50 mg/Kg body weight, once a day for 3 days, each injection spaced apart by 3–4 days), placed in a stereotaxic frame and exposed to cranial irradiation using a Siemens Stabilopan x-ray system operated at 300 kVp and 20 mA. Animals were protected with a lead shield that covered the entire body, but left unshielded a 3.22 × 11-mm treatment field above the hippocampus (interaural 3.00 to 0.00) exposed to x-ray. Dosimetry was done using a Capintec Model PR06G electrometer ionization chamber and Kodak Readypack Radiographic XV films. The corrected dose rate was approximately 1.8 Gy per min at a source to skin distance of 30 cm. The procedure lasted 2 min and 47 s, delivering a total of 5 Gy. Three 5 Gy doses were delivered on days 1, 4, and 8.

### Voltage sensitive dye imaging of evoked neural activity

NCff female mice were induced with vehicle or TAM and processed for VSDI 8 weeks later. x-irradiated C57BL/6J mice were processed for VSDI 14 weeks post targeted x-irradiation. To prepare living brain slices, animals were deeply anesthetized with Nembutal (>100 mg/kg, i.p.), transcardially perfused with artificial cerebrospinal fluid (CSF), rapidly decapitated, and their brains removed. Hippocampal slices were cut 400 μm thick with a vibratome (VT1200S; Leica Systems, Germany) in the artificial cerebrospinal fluid (CSF) (in mM: 126 NaCl, 2.5 KCl, 26 NaHCO3, 2 CaCl2, 2 MgCl2, 1.25 NaH2PO4, and 10 glucose) with a broad-spectrum excitatory amino acid antagonist kynurenic acid (0.3 mM). Slices were first incubated in the ACSF for 30 min to 1 h at 32°C, and after the initial incubation period, transferred to a chamber containing ACSF with 0.02 mg/ml of an oxonol dye, NK3630 for the dye staining at room temperature. The dye was chosen because of its good sensitivity, low bleaching and phototoxicity, as well as its preferential staining of neurons with a low affinity for glial cells (Konnerth et al., 1987; Jin et al., 2002). The NK3630 dye has been characterized in previous studies and has its peak signal-to-noise ratio centered at the trans-illumination of 705 nm (Jin et al., 2002). Slices were stained for 1 h and then maintained in regular ACSF before use. Throughout the incubation, staining and recording, slices were continuously perfused with 95% O_2_-5% CO_2_.

The laser scanning photostimulation and imaging system is described in detail previously (Xu et al., 2010; Olivas et al., 2012). Slices were visualized with an upright microscope (BW51X; Olympus,Tokyo, Japan) with epi-fluorescent and infrared differential interference contrast optics. Electrophysiological recordings, photostimulation, and imaging of the slice preparations were done in a slice perfusion chamber mounted on a motorized stage of the microscope. At low magnification (2× objective lens), laminar and cytoarchitectonic features of brain slices were visualized under infrared bright-field transillumination; and the slice images were acquired by a high resolution digital CCD camera (Retiga 2000, Q-imaging Inc, Austin, TX). Digitized images from the camera were used for guiding and registering stimulation sites in hippocampal slices. A bipolar electrode (FHC Inc, Bowdoin, ME) was used to deliver electrical stimulation in DG apex. For laser photostimulation via glutamate uncaging, MNI-caged-l-glutamate (4-methoxy-7-nitroindolinyl-caged l-glutamate, Tocris Bioscience, Ellisville, MO) was added to 20–25 ml of circulating ACSF for a concentration of 0.4 mM caged glutamate. A laser unit (model 3501; DPSS Lasers, Santa Clara, CA) was used to generate 355 nm UV laser for glutamate uncaging. The laser beam was directed through the optical path of our system and physical size of laser excitation in the slice was about 200 μm in a Gaussian profile. Short durations of laser flashes (i.e., 1–3 ms) were controlled using an electro-optical modulator (i.e., pockels cell) and a mechanical shutter. Various laser stimulation positions were achieved through galvanometers-driven XY scanning mirrors, as the mirrors and the back aperture of the objective were in conjugate planes, translating mirror positions into different scanning locations at the objective lens focal plane. A dual camera port was used to couple the Q-imaging camera and the laser scanning photostimulation system to a MiCAM02 fast imaging system (SciMedia USA Ltd, Costa Mesa, CA) for voltage-sensitive dye (VSD) imaging. Optical recording of VSD signals was performed by the MiCAM02 system with a sampling rate of 2.2 ms per frame [frame resolution 88 (w) × 60 (h) pixels]. The imaging field covered the area of 2.56 × 2.14 mm^2^ with a spatial resolution of 29.2 × 35.8 μm/pixel. Trials were obtained every 8 s and the recording periods were 1000 frames for stimulation trial. For both electrical and photostimulation experiments, data averaging of 4 trials was used for quantification, and VSD images were smoothed by convolving images with a Gaussian spatial filter (kernel size: 5 × 5 pixels; δ size: 1 × 1 pixel) and a Gaussian temporal filter (kernel size: 3 frames; δ size: 1 frame). VSD signal amplitudes were expressed as standard deviation (SD) multiples above the mean baseline signal for display and quantification. Larger values conveyed stronger responses. Image quantification and measurements were performed with custom-made Matlab Programs.

**Figure 1:**
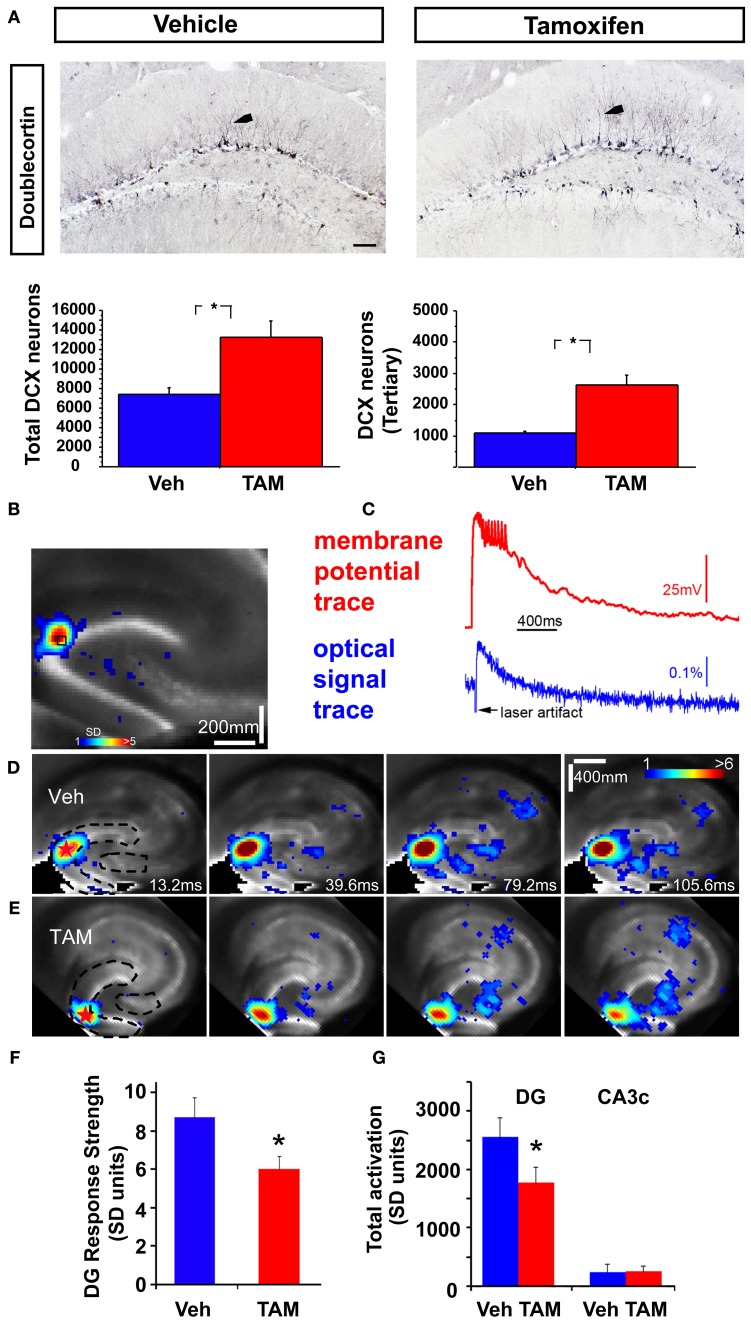
**Increasing adult hippocampal neurogenesis decreases photostimulation evoked DG excitability without affecting CA3c activation. (A)** Representative DCX immunostained coronal hippocampal sections of Veh and TAM treated NCff mice. Arrows in insets indicate DCX neurons with at least tertiary dendrites. Quantification of DCX population. Total DCX+ neurons: Veh: 7435.5 ± 629.2, TAM: 13228.5 ± 1720.2, Mean ± SEM, *p* = 0.0195, *n* = 4/gp, DCX expressing neurons with tertiary dendrites: Veh: 1089.0 ± 55, TAM: 2623.5 ± 309.2, Mean ± SEM, *p* = 0.0027, *n* = 4/gp. Scale bar: 100 μm. **(B,C)** VSD imaging and simultaneous whole cell recording indicate that photostimulation evoked VSD signals are closely related to membrane potential depolarization of individual neurons. The measurements are taken from a DG site of a wild type C57BL/6J mouse slice, as indicated by the small black square, in response to spatially restricted photostimulation. The photostimulation locations are labeled with red stars. The VSD image frame in **(B)** is plotted beginning at the peak membrane depolarization of the recorded neuron. Color-coded activity is superimposed on the background slice image. The color scale codes VSD signal amplitude expressed as SD (standard deviation) multiples above the mean baseline. Warmer colors indicate greater excitation. **(C)** Shows the aligned optical signal trace [VSD signal in the percent change of pixel intensity (ΔI/I%)] and current-clamp recording trace. **(D,E)** Time series data of VSD imaging of photostimulation-evoked circuit activity in example slices from Veh and TAM treated NCff mice, respectively. The stimulation site in DG is indicated by a red star; the image frames are labeled with the specified times after the stimulation onset. Dashed lines in first panels of **(D,E)** indicate delineated regions for measurement of VSD activity. **(F)** Average DG response strength at the photostimulation sites between Vehicle and TAM treated NCff mice (*n* = 7 mice per group). Overall mean: Veh: 8.72 ± 1.01, *n* = 7 slices, TAM: 5.98 ± 0.70 (mean ± SE SD units), *n* = 8 slices, *p* < 0.05. **(G)** DG activation and output response. Total DG responses (summed amplitudes of activated pixels across 10 peak response frames): Veh: 2552 ± 346 and TAM, 1766 ± 279.2, *n* = 7 slices each, *p* < 0.05, (mean ± SE SD units). The proximal CA3 (CA3c) activation followed with DG photostimulation did not differ between the groups. Total response values: Veh: 257.5 ± 94.7 and TAM: 235.8 ± 138.6 (mean ± SE SD units). ^*^p < 0.05.

For quantitative analysis of evoked activation in image frames, the mean and standard deviation of the baseline activity of each pixel across the 50 frames preceding photostimulation was first calculated, and then activated pixels were measured. The activated pixel was empirically defined as the pixel with the amplitude ≥1 SD above the mean of the corresponding pixel's amplitude preceding the stimulation (equivalent to the detectable signal level in the original VSDI maps of ΔI/I%). As illustrated in Figures 1D,E, we manually delineated the contours of blades of DG and region of interest for CA3c using slice background images. The total activation of evoked VSD responses was measured from these delineated regions across 10 peak response frames. The hilus was excluded from analysis.

For statistical comparisons between groups, the data were checked for normality distribution and equal variance. If the criteria where met, statistical significance was assessed by unpaired two-tailed student's *t*-tests or analysis of variance (ANOVA); when the criteria were not met, a Mann–Whitney *U*-test was used. Analysis of stimulation strength intensities was performed using repeat measures ANOVA. In all experiments, ^*^corresponds to a *p*-value of <0.05.

### Whole cell mIPSC recording and data analysis

Mice were decapitated after anesthetized with isoflurane vapor. Both hippocampi were rapidly removed and 300 μm transverse slices were cut in ice-cold dissection buffer (in mM: 212.7 sucrose, 2.6 KCl, 1.23 NaH2PO4, 26 NaHCO3, 10 dextrose, 3 MgCl2, and 1 CaCl2, saturated with 5% CO2/95% O2) with a vibratome (Vibratome 3000 series). The slices were then transferred to a holding chamber filled with artificial cerebrospinal fluid (ACSF, in mM): 124 NaCl, 5 KCl, 1.25 NaH_2_PO_4_, 26 NaHCO_3_, 1.5 MgCl_2_, and 2.5 CaCl_2_, saturated with a mixture of 5% CO_2_/95% O_2_. The slices were recovered for at least 1 h in room temperature before recording. Slices were transferred to a submersion chamber perfused with oxygenated ACSF. Miniature IPSC (mIPSC) was pharmacologically isolated by adding 100 μM DL-APV, 10 μM NBQX, and 1 μM TTX in the bath. An upright microscope (Nikon E600FN) equipped with infrared oblique illumination was used to visualize and identify the mature dentate granule cells based on both the shape and location of the soma. Neurons were patched with glass electrode (3–5 MΩ resistance) filled with CsCl-based internal solution (in mM): 120 CsCl, 8 KCl, 10 EGTA, 10 HEPES, 10 QX-314, and 0.1% biocytin. This internal solution allows recording of mIPSCs as inward current at negative holding potentials (Gao et al., 2010). The neurons were held at −80 mV and responses were recorded by an Axon Multiclamp 700B amplifier (Molecular Devices), digitized at 10 kHz through a data acquisition board (National Instruments), and acquired using a custom-made Igor Pro software (WaveMetrics). The identity of the DG cell was further confirmed by morphology with *post-hoc* biocytin staining and imaging. The miniature events were analyzed by MiniAnalysis program (Synaptosoft). The detection threshold was set to be three times of the RMS noise. Events were discarded if the rise time was greater than 5 ms. Only cells with ≤25 MΩ access resistance and ≥300 MΩ input resistance were used. Student's *t*-test was used for comparison of average amplitude and frequency between the two groups, and Kolmogorov-Smirnov test was used for comparing the cumulative probability. *P* < 0.05 was take as statistically significant. Data are expressed as mean ± standard error (SE).

### Retroviral vectors, virus preparation and injections

A stable human 293-derived retroviral packaging cell line (kindly provided by S. Ge, Stony Brook University School of Medicine, NY) was co-transfected with CAG-GFP-IRES-CRE retroviral vector (Jagasia et al., 2009) (generous gift of Dr. D. C. Lie) and pVSVG by calcium phosphate-mediated transfection. Virus-containing supernatant was harvested 36, 48, and 60 h after transfection and concentrated by ultracentrifugation at 25,000 rpm for 1.5 h. Adult (8 weeks old) *Bax ^f/f^* mice and *Bax* ^+/+^ littermates were maintained under standard housing conditions, and following anaesthetization, equal volume of viruses were stereotaxically and bilaterally injected into the dorsal DG (1 μ l per site at 0.1 μ l/min) using the following coordinates: anterioposterior = −2 mm from bregma; lateral = ± 1.6 mm; ventral = 2.5 mm. Injection needles were left in place for an additional 10 min after injection to ensure even distribution of the virus. Mice were sacrificed 4 weeks post infection to examine mossy fiber terminal (MFT)-interneuron connectivity of GFP+ adult-born neurons that had undergone recombination for Bax or just expressed GFP.

### Immunohistochemistry

Mice were anesthetized with ketamine/xylazine (100 and 7 mg/kg, respectively) and transcardially perfused (cold saline, followed by 4% cold paraformaldehyde in PBS). Brains were post fixed overnight in 4% paraformaldehyde at 4°C, then cryoprotected in 30% sucrose, and stored at 4°C. Forty micrometres coronal serial sections of the entire hippocampus using a cryostat and stored in PBS with 0.01% sodium azide. For Doublecortin (DCX) immunohistochemistry on NCff mice, floating sections were first quenched to remove endogenous peroxidase activity (1% H_2_O_2_ in PBS:Methanol). Sections were then washed in PBS, blocked (PBS containing 0.3% triton and 10% NDS) and incubated with primary antibody overnight at 4°C (DCX, goat, 1:500, SantaCruz, CA). Following washes in PBS, sections were incubated with horse radish peroxidase coupled biotinylated secondaries. Following incubation with ABC solution (Vector, Burlingame, CA), the color reaction was carried out using a DAB kit (Vector, Burlingame, CA). For Doublecortin immunohistochemistry on x-irradiated mice, sections were washed 3 times in PBS, blocked in PBS buffer containing 0.3% triton and 10% NDS and incubated in primary antibodies overnight shaking at 4°C (DCX, goat, 1:500, SantaCruz, CA). The next day sections were washed 3 times in PBS and incubated with fluorescence coupled secondary antibodies (Jackson ImmunoResearch, West Grove, PA) for 2 h at RT. An unbiased and blinded quantification protocol was used to quantify Doublecortin positive cells in the GCL of the DG along the septo-temporal axis (Sahay et al., 2011b).

For VGAT and GAD-67 immunohistochemistry, sections were washed 3 times in PBS, blocked in PBS buffer containing 3% NDS (VGAT) or 0.2% (GAD-67) and incubated in primary antibodies overnight in PBS buffer containing 0.3% triton (VGAT, rabbit, 1:500, Synaptic systems) or PBS (GAD-67, Millipore, MAB5406, 1:1000) shaking at 4°C. The next day sections were washed 3 times in PBS and incubated with fluorescence coupled secondary antibodies (Jackson ImmunoResearch, West Grove, PA) for 2 h at RT. For GFP, GAD-67 and VGLUT1 immunohistochemistry, sections were washed 3 times in PBS, blocked in PBS buffer containing 10% NDS and 0.3% triton for 2 h and then incubated in primary antibodies overnight in PBS buffer containing 0.3% triton (GFP, rabbit, 1:500, Invitrogen; GAD-67, mouse, 1:1000;Millipore; VGLUT1, Guinea pig, 1:3000;Synaptic systems) shaking at 4°C. The next day, sections were washed 3 times in PBS and incubated with fluorescence coupled secondary antibodies (Jackson ImmunoResearch, West Grove, PA) for 2 h at RT.

For biocytin processing and imaging after recording, hippocampal slices were fixed in 4% paraformaldehyde overnight at 4°C. Slices were then rinsed twice, 10 min each, in 0.1 M phosphate buffer (PB: 19 mM NaH_2_PO_4_·H2O, and 81 mM Na_2_HPO_4_) at room temperature and permeabilized in 0.1 M PB with 2% Triton X-100 for 1 h. Slices were the incubated in 1% Triton X-100–0.1 M PB containing 1:2000 avidin-AlexaFluor 488 conjugate overnight at 4°C. Slices were rinsed twice in 0.1 M PB, 10 min each, before mounted on precleaned glass slides and coverslipped using a mounting solution (ProLong antifade; Invitrogen).

### Image analysis

For quantification of VGAT and GAD67 immunostaining intensity, confocal z-stack images were acquired with a Nikon A1R Si confocal laser, a TiE inverted research microscope, and NIS Elements software (Nikon Instruments, 1300; Walt Whitman Road, Melville, N.Y. 11747-3064) of the DG along the septotemporal axis of the hippocampus axis. Images were taken using a 20× objective at a resolution of 1024 × 1024 pixels (0.31 μm/px), pixel dwell time was 0.5 μm/s, and line averaging was set to 4. Z-stacks were 20 μm thick, had a step size of 1 μm, and were converted to maximum intensity projections before analysis. Mean immunofluorescence intensity measurements for the hilus, GCL, and molecular layers of the DG were calculated as a percentage of background labeling in fimbria/fornix.

For quantification of GFP+ MFT contacts with GAD67+ processes in the hilus, confocal z-stack images were acquired using a Nikon A1R Si confocal laser, a TiE inverted research microscope, and NIS Elements software (Nikon Instruments, 1300; Walt Whitman Road, Melville, N.Y. 11747-3064). Images of the DG along the septotemporal axis of the hippocampus axis were taken using a 60× objective at a resolution of 1024 × 1024 pixels (0.31 μm/px) with pixel dwell time set at 0.5 μm/s and line averaging at 2. Three-dimensional Z-series stacks were captured at 0.5 μm increments with six to eight times optical zoom. At least 70 GFP+ MFTs were analyzed per brain.

## Results

To address how changes in levels of adult-hippocampal neurogenesis affects excitability of the DG, we combined VSDI with two previously characterized mouse models, one in which adult hippocampal neurogenesis is genetically and inducibly enhanced and one in which adult-born dentate granule neurons are ablated. We employed high-speed VSDI because it allows quantitative analysis of circuit-level neural activity on millisecond time scales with micrometer spatial resolution and a field of view spanning entire cortical networks (Airan et al., 2007; Xu et al., 2010; Coulter et al., 2011; von Wolff et al., 2011; Yu et al., 2013). To genetically increase the number of adult-born dentate granule neurons, we induced recombination of *Bax* in adult neural stem cells as done previously using *iBax^nestin^* mice (Sahay et al., 2011b). These mice show increased survival and functional integration of adult-born dentate granule neurons. Analysis of numbers of young-adult born dentate granule neurons 8 weeks following vehicle or TAM treatment by doublecortin (DCX) immunohistochemistry showed a 1.8 fold increase in the number of DCX expressing neurons and a 2.4 fold increase in DCX expressing neurons with tertiary dendrites (Figure 1A). Thus, at 8 weeks following TAM treatment, *iBax^nestin^* mice show a persistent expansion in the pool of DCX expressing neurons. Consistent with our previous and other studies (Carlson and Coulter, 2008; Xu et al., 2010), alignment of the VSD signal evoked by photostimulation via glutamate uncaging in the GCL with electrical signals from simultaneous whole cell recordings showed faithful tracking of the optical signal trace with that of membrane potential trace (Figures 1B,C). We had previously shown that the photostimulation-evoked VSD responses were mediated by glutamate and its receptors and that VSD responses to glutamate uncaging were essentially abolished by the ionotropic glutamate receptor antagonists (CPP and CNQX) (Xu et al., 2010). Furthermore, reducing neurotransmitter release and synaptic spread from stimulated neurons using low Ca2+ and high Mg2+ ACSF solution restricted VSD changes to the region proximal to the stimulation site (Xu et al., 2010). This indicates that most of the neural activity distal to the stimulation site reflects synaptic spread of activity to postsynaptic neurons, rather than retrograde spread of activity to distant dendrites of directly stimulated cells. We had previously confirmed spatial precision of laser photostimulation by recording from a single site in response to laser stimulation at varying distances from the recording site. Only neurons within or close to the photostimulation site were activated or exhibited action potentials (Antonio et al., 2013). Here, we recorded the VSDI signal and examined time series of photostimulation-evoked activity propagation in the DG and CA3c of *iBax^nestin^* mice and controls (Figures 1D,E). The color scale for VSD images codes the strength of excitatory activity (reflecting membrane potential depolarization of neuronal ensembles) with warmer colors indicating greater excitation. Comparison of average DG response strength showed a significant decrease in the *iBax^nestin^* mice relative to controls (Figure 1F) and a reduction in spread of propagation of VSD signal in the DG (Figure 1G). No significant change in VSD signal propagation to CA3c between the two groups was observed (Figure 1G).

**Figure 2:**
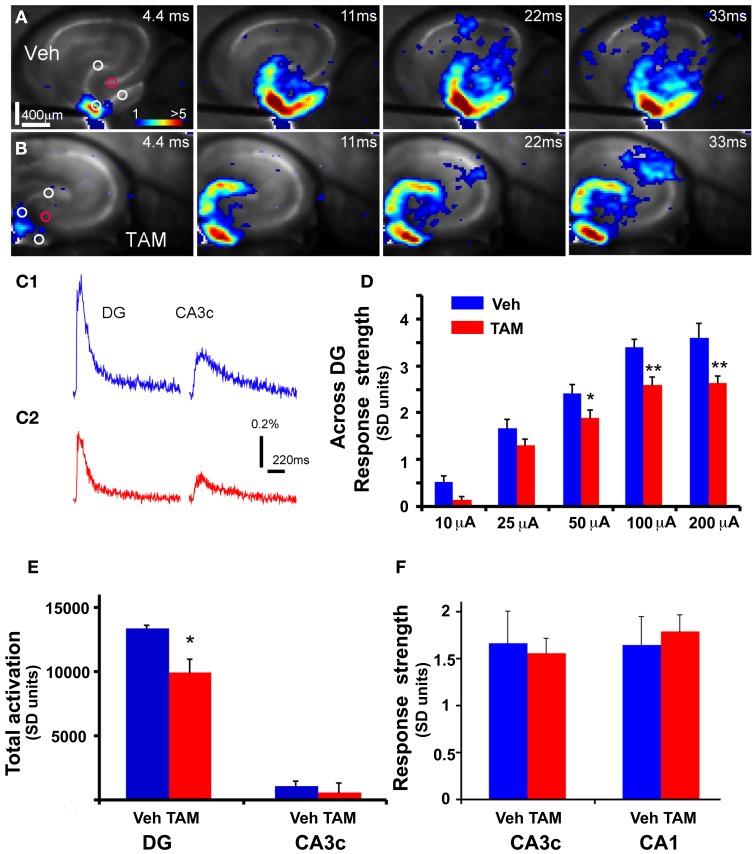
**DG of *iBax^nestin^* mice shows decreased strength of neuronal activation following electrical stimulation. (A)** Time series data of VSD imaging of hippocampal circuit activity in response to electrical stimulation (100 μA, 1 ms) at DG with a bipolar extracellular stimulation electrode in a Vehicle treated NCff mouse slice. The color scale codes VSD signal amplitude expressed as SD multiples above the mean baseline. Warmer colors indicate stronger excitatory responses. **(B)** Time series data of VSD imaging of the circuit activity in response to DG electrical stimulation (100 μA, 1 ms) in a TAM treated NCff mouse slice. **(C1)** The time courses of VSD signal [in the percent change of pixel intensity (ΔI/I%)] from the regions indicated by the small circles in the first frame in DG apex and CA3c of **(A)**, respectively. **(C2)** The time courses of VSD signal from the small regions indicated in the first frame in DG apex and CA3c of **(B)**. **(D)** Comparisons of the average VSD response strength across DG (measured from 3 regions indicated by the three white circles at the DG granule cell layer) to electrical stimulation in the DG center of different amplitudes for the control and experimental groups. The data points (mean ± SE) are from 7 mice (22–26 slices total) each group, calculated for each slice and averaged for each animal. Because of slice conditions, responses for all stimulation intensities were not obtained from all slices. Two-Way repeated measures ANOVA, (treatment) *F*_(1, 4)_ = 10.5, *p* = 0.03. ^*^p ≤ 0.05, ^**^p ≤ 0.01. **(E)** VSD response strength of CA3c (delineated by red circle) to DG electric stimulation (100 μA) between Vehicle and TAM treated NCff mice. **(F)** Total DG and CA3c activation (summed amplitudes of activated pixels across 10 peak response frames) in response to DG electric stimulation (100 μA) between Vehicle and TAM treated NCff mice.

**Figure 3:**
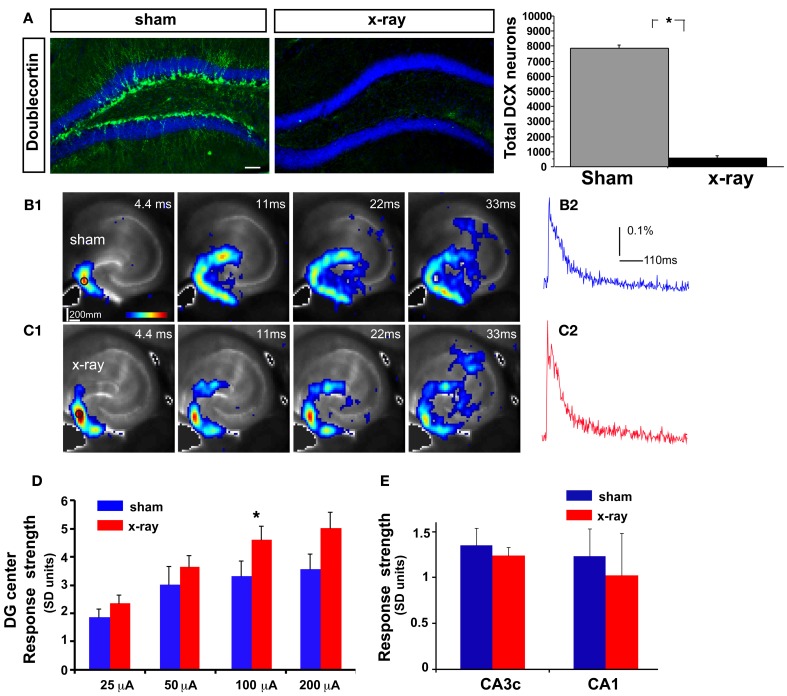
**DG excitability is increased in mice in which adult hippocampal neurogenesis is ablated. (A)** Representative DCX immunostained coronal hippocampal sections of sham and x-irradiated mice. Total DCX counts: sham, 7873 ± 176.9, x-ray, 564.6 ± 148.9, Mean ± SEM, *n* = 3/gp, *p* < 0.0001. **(B1–C1) (B1)** and **(C1)** show time series data of VSD imaging of hippocampal circuit activity in response to electrical stimulation (100 μA, 1 ms) at DG with a bipolar extracellular stimulation electrode in sham and x-rayed mouse slices, respectively. **(B2)** and **(C2)** display the time courses of VSD signal [in the percent change of pixel intensity (ΔI/I%)] from the regions indicated by the small circles in the first frame in the DG center in **(B1)** and **(C1)**, respectively. **(D)** Comparisons of the average peak strength of VSD response to electrical stimulation at center of DG of different amplitudes for the control and experimental groups. The data points (mean ± SE) are from 6 to 8 mice in each group (18–21 slices total), calculated for each slice and averaged for each animal. Because of slice conditions, responses for all stimulation intensities were not obtained from all slices. VSD response strength to DG electric stimulation (100 μA) differed significantly between sham and x-irradiated slices, *p* < 0.05. Analysis across all stimulation intensities showed a trend toward increased cellular activation in x-irradiated mice. Two-Way repeated measures ANOVA, (treatment) *F*_(1, 11)_ = 2.7, *p* = 0.12, (stimulation intensity × treatment) *F*_(3, 33)_ = 1, *p* = 0.4. **(E)** Total DG and CA3c activation (summed amplitudes of activated pixels across 10 peak response frames) in response to DG electric stimulation (100 μA) between sham and x-irradiated groups. ^*^p <0.05.

While laser scanning photostimulation affords spatially restricted activation, it does not permit assessment of response strength in the DG as a function of stimulation intensities that is achievable with electric stimulation. To determine whether increasing the number of adult-born dentate granule neurons alters VSD response strength in the DG, we performed VSDI on brain slices from *iBax^nestin^* mice and controls following electrical stimulation within the GCL of the DG. Comparisons of average VSD response strength across the DG as measured from three different regions within the GCL showed a significant reduction in *iBax^nestin^* mice relative to controls in response to a range of stimulation intensities (Figures 2A–D). VSD response strength at DG center also showed lower activation in *iBax^nestin^* mice relative to controls [Two-Way repeated measures ANOVA, (treatment) *F*_(1, 4)_ = 11.6, *p* = 0.02]. Consistent with the laser photostimulation VSDI analysis, *iBax^nestin^* mice showed a reduction in VSDI signal strength in the DG (Figure 2E). VSD response strength in CA1 and CA3c remained unchanged following 100 μ A DG stimulation (Figure 2F). Together, these studies suggest that genetically increasing the number of adult-born dentate granule neurons in the DG decreases activation strength of dentate granule neurons in response to photostimulation and electrical stimulation and restricts spread of neural activity in DG while maintaining propagation of activity to CA3c. To determine whether blockade of adult hippocampal neurogenesis alters the magnitude of activation in the DG, we performed VSDI on brain slices from hippocampal x-irradiated mice and controls following electrical stimulation within the GCL of the DG. x-irradiation resulted in almost complete ablation of adult-born neurons as evidenced by loss of DCX expressing cells (Figure 3A). Although responses obtained from x-irradiated C57BL/6J slices showed more variability than slices from *iBax^nestin^* mice, average VSD response strength at DG center showed significantly greater activation in hippocampal x-irradiated mice compared with sham treated mice at 100 μA stimulation (*p* < 0.05), (Figures 3B–D). Average VSD response strength across the DG of hippocampal x-irradiated mice and sham C57BL/6J mouse slices were largely similar [Two-Way repeated measures ANOVA, (treatment) *F*_(1, 11)_ = 2.3, *p* = 0.15]. The Total DG and CA3c activation in response to DG electric stimulation (100 μA) between sham and x-irradiated groups was not different (Figure 3E). These data lend further support to the notion that levels of adult neurogenesis modify DG excitability.

**Figure 4:**
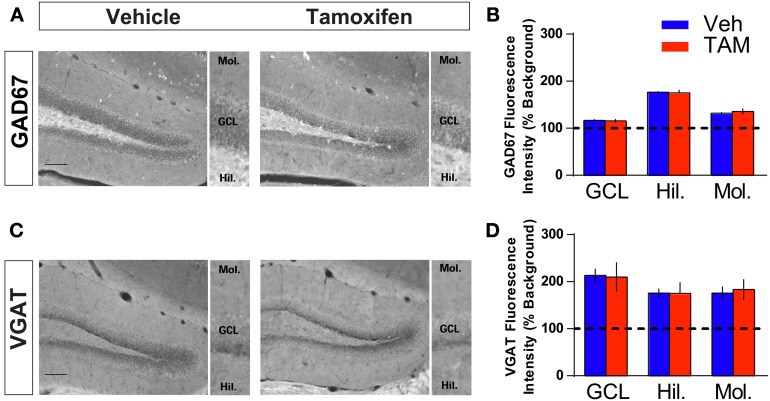
**Increasing adult hippocampal neurogenesis does not affect presynaptic GABAergic innervation of mature dentate granule neurons. (A,B)** GAD67 immunohistochemistry and quantification of GAD67 levels in (GCL), hilus and molecular layers of DG (inset). NCff (Veh and TAM) mice show similar levels of GAD67 in the DG. **(C,D)** VGAT immunohistochemistry and quantification of VGAT levels in GCL, hilus and molecular layers of DG (inset). NCff (Veh and TAM) mice show similar levels of VGAT in the DG. Scale bar: 500 μm.

**Figure 5:**
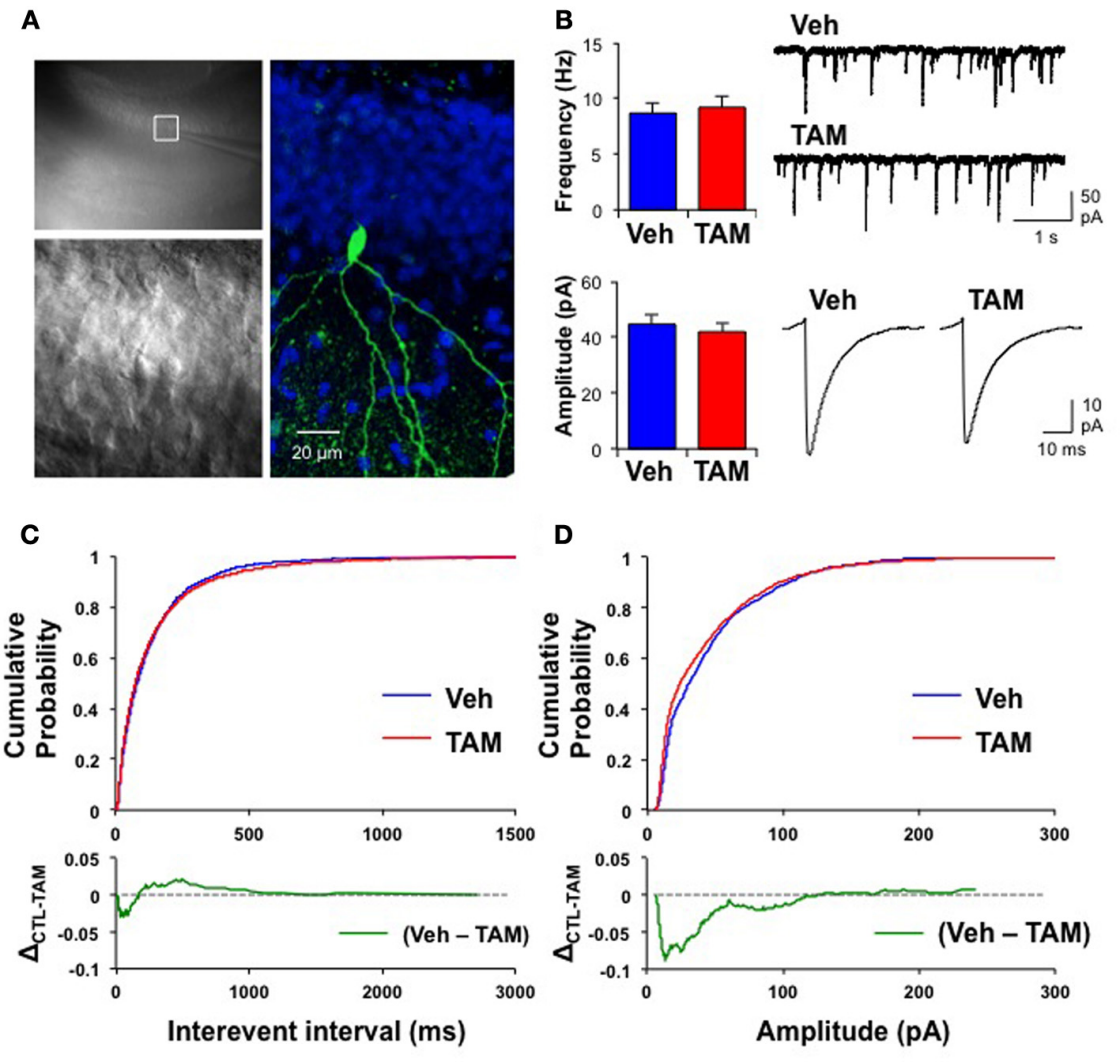
**Mature dentate granule neurons in *iBax^nestin^* mice show redistribution of inhibitory synaptic weights without changes in pattern of mIPSC generation. (A)** Recording from a representative mature dentate granule cell. Left, top: A low magnification image showing a patch electrode on a neuron located in the outer blade. The area outlined by the white square is shown below in higher magnification. Right: The same neuron processed for biocytin, which was placed inside the recording pipette. Green: biocytin, Blue: DAPI. **(B)** No significant difference in the average mIPSC frequency (top left, Veh: 8.7 ± 0.89 Hz; TAM: 9.2 ± 1.03 Hz; *t*-test: *p* = 0.71) and the average mIPSC amplitude (bottom left, Veh: 45 ± 3.3 pA, *n* = 11; TAM: 42 ± 3.2 pA, *n* = 20; *t*-test: *p* = 0.57) between control and *iBax^nestin^* mice. Top right: Representative mIPSC traces. Bottom right: Average mIPSC traces. There was no difference in the kinetics of the mIPSCs between the two groups (10–90 rise: Veh: 1.9 ± 0.07 ms; TAM: 1.9 ± 0.05 ms, *p* = 0.85; decay time constant: Veh: 6.1 ± 0.45 ms; TAM: 6.5 ± 0.28 ms, *p* = 0.41). **(C)** No change in pattern of mIPSC generation between two groups. Top: Cumulative probability of mIPSC interevent intervals from control and *iBax^nestin^* mice. No significant difference between the two groups (Kolmogorov-Smirnov test: *p* = 0.1). Bottom: Subtraction of the two cumulative probability graphs (Veh–TAM) confirms a minimal change in the distribution of mIPSCs interevent intervals. **(D)** Changes in the distribution of inhibitory synaptic weight in *iBax^nestin^* mice Top: Cumulative probability of mIPSC amplitudes from control and *iBax^nestin^* mice. There was a significant difference between the two groups (Kolmogorov-Smirnov test: *p* < 0.001). Bottom: Subtraction of the two cumulative probability graphs (Veh–TAM) reveals a significant increase in the fraction of smaller and larger mIPSCs in the TAM treated group.

Because of the placement of stimulation electrodes and location of glutamate uncaging in the GCL, the effects on VSDI of DG activation of *iBax^nestin^* and hippocampal x-irradiated mice are likely to be due to changes in local circuitry that mediates feed back inhibition onto the DG. Increased feedback inhibition onto the DG in *iBax^nestin^* mice may arise from increased inhibitory inputs onto mature dentate granule neurons or increased excitatory drive or synapses of young-adult born neurons onto hilar interneurons. We first tested whether *iBax^nestin^* mice exhibited increased inhibitory inputs onto mature dentate granule neurons in two complementary ways. First, we examined immunoreactivity for the GABAergic presynaptic marker, vesicular GABA transporter, VGAT and glutamic acid decarboxylase, GAD67, in the GCL, hilus and molecular layer of the DG in *iBax^nestin^* mice and controls by confocal microscopy. No difference in VGAT or GAD67 immunoreactivity was observed between the two groups (Figures 4A–D). Second, to assess functional alteration of the inhibitory inputs onto the mature dentate granule cells, we recorded mIPSCs from mature dentate granule neurons exclusively in the outer third of the GCL, which is primarily populated by mature dentate granule neurons (Esposito et al., 2005; Laplagne et al., 2006), of DG of *iBax^nestin^* mice and controls (Figure 5A). Events were discarded if the rise time was greater than 5 ms. There was no significant difference in the RMS noise (Veh = 2.67 ± 0.19, *n* = 11; TAM = 2.44 ± 0.08, *n* = 20; *p* = 0.29), input resistance (Veh = 527 ± 47 MΩ, TAM = 498 ± 45 MΩ, *p* = 0.62) or the series resistance (Veh = 24 ± 0.5 MΩ, TAM = 24 ± 0.5 MΩ, *p* = 0.66) between the two groups. mIPSCs were recorded as inward currents at hyperpolarizing holding potentials using a symmetrical Cl^−^ gradient (Gao et al., 2010). We did not observe any significant difference in the average amplitude or the average frequency of mIPSCs between *iBax^nestin^* mice compared to controls (Figure 5B). We then examined the cumulative probability distribution of amplitudes of mIPSCs (Kilman et al., 2002; Gao et al., 2010). Interestingly, the cumulative probability distribution of amplitudes of all mIPSCs recorded from *iBax^nestin^* mice was statistically significantly different from those of controls (Figure 5D), which suggests a change in the distribution of inhibitory postsynaptic strength. Specifically, *iBax^nestin^* mice showed more smaller and larger mIPSCs (Figure 5D, bottom panel) indicative of a redistribution of inhibitory synaptic weights. In contrast, we did not observe a significant difference in the cumulative probability distribution of interevent interval of mIPSCs (Figure 5C) suggesting that there is no change in the pattern of mIPSC generation. Together with a lack of a change in the average mIPSC frequency, this suggests that the number of inhibitory synaptic contacts and/or presynaptic function is unaltered in *iBax^nestin^* mice.

**Figure 6:**
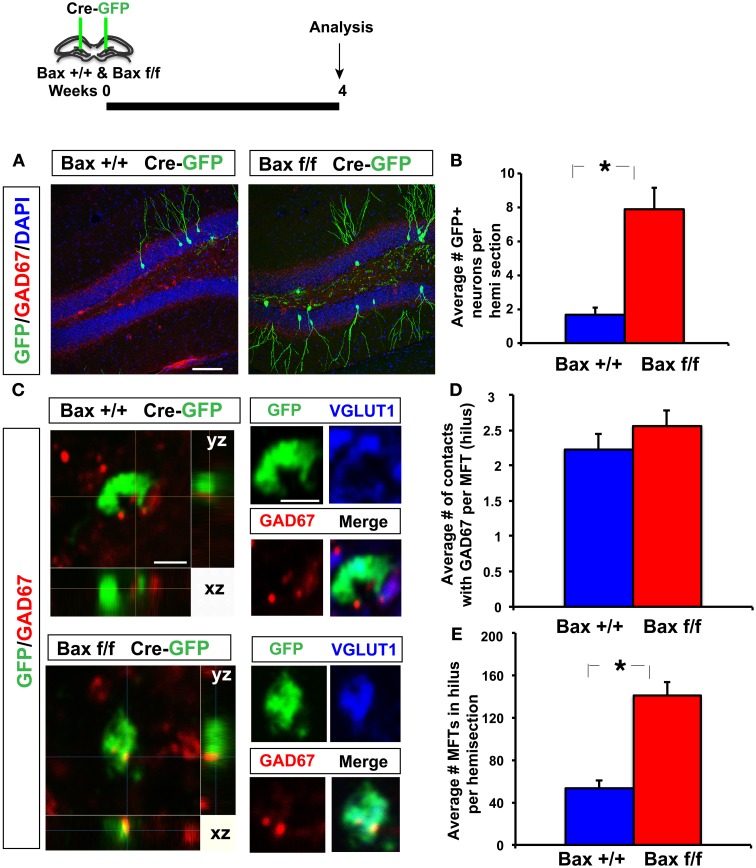
**Inducible recombination of *Bax* in progenitors in adult DG increases the number of excitatory synaptic contacts of adult-born dentate granule neurons with hilar interneurons. (A,B)** Representative images of DG of *Bax ^+/+^* and *Bax ^f/f^* littermates processed for GAD67, GFP, and DAPI and quantification showing increased number of 4 weeks old GFP+ adult-born dentate granule neurons in *Bax ^f/f^* mice. **(C)** Confocal images in xy, xz, and yz planes showing GFP+ contacts of small MFT with GAD67+ processes in hilus of *Bax ^+/+^* and *Bax ^f/f^* mice. **(D)** DG of *Bax ^f/f^* mice (140.7 ± 12.9) show a significant increase in GFP+ MFTs in hilus compared to *Bax ^+/+^* littermates (53.16 ± 7.24) (*n* = 12 hemisections from 2 *Bax ^+/+^* mice, *n* = 17 hemisections from 3 *Bax ^f/f^* mice, *p* < 0.001, unpaired *t*-test). **(E)** Hilus of *Bax ^f/f^* (2.55 ± 0.22) and *Bax ^+/+^* (2.22 ± 0.21) littermates show equivalent number of contacts with GAD67 processes per MFT (*n* = 75 MFTs from 2 *Bax ^+/+^* mice and *n* = 70 MFTs from 3 *Bax ^f/f^* mice, *p* = 0.27, unpaired *t*-test). Scale bar: 100 μ m **(A)**, 2 μ m **(C)**. Results are Mean ± SE. ^*^p <0.05.

To determine changes in the anatomical basis of synaptic connectivity between adult-born dentate granule neurons and hilar interneurons, we injected an identical volume of retroviruses co-expressing Cre recombinase and GFP bilaterally into the DG of adult *Bax* ^+/+^ and *Bax ^f/f^* littermates to infect equivalent numbers of dividing progenitors. We then quantified the number of GFP+ synaptic contacts of 4 weeks old adult-born neurons with hilar interneurons. We chose 4 weeks post infection as young-adult born neurons exhibit heightened synaptic plasticity (Schmidt-Hieber et al., 2004; Ge et al., 2007), insensitivity to GABAergic inhibition (Wang et al., 2000; Snyder et al., 2001; Esposito et al., 2005; Overstreet Wadiche et al., 2005; Saxe et al., 2006; Ge et al., 2008; Massa et al., 2011; Sahay et al., 2011b; Marin-Burgin et al., 2012) and have passed a *Bax* dependent cell death window (Sahay et al., 2011b) at this stage of maturation. As expected, we observed a significant increase in the number of GFP+ 4 weeks old dentate granule neurons in adult *Bax ^f/f^* mice because of cell-autonomous recombination of Bax and enhanced survival of GFP+ dentate granule neurons (Figures 6A,B). Analysis of GFP+ synaptic contacts of adult-born dentate granule neurons onto GAD67+ processes of hilar interneurons revealed that they were primarily located within small MFTs (Acsady et al., 1998). Confocal analysis revealed that most of these GFP+ MFTs contained vesicular glutamate transporter-1 (VGLUT1) indicative of functional excitatory synapses (58 of 77 GFP+ MFTs, *Bax* ^+/+^ and 56 of 78 GFP+ MFTs, *Bax ^f/f^*) (Figure 6C). Moreover, within small GFP+ MFTs, the average number of synaptic contacts with hilar GAD67+ processes was similar in DG of *Bax* ^+/+^ and *Bax ^f/f^* mice (Figures 6C,D). However, *Bax ^f/f^* mice showed a significant increase in the average number of small MFTs contacting GAD67+ processes in hilus. This suggests that Cre dependent recombination of *Bax* in progenitors in adult DG increased number of excitatory synapses onto hilar interneurons (Figure 6E) by enhancing survival of more adult-born dentate granule neurons and therefore, potentially, increasing excitatory drive onto hilar interneurons.

## Discussion

Studies in rodents have implicated adult-born neurons as important for pattern separation functions of the DG. While the electrophysiological properties of adult-born neurons have been extensively studied, much less is known about how adult-born neurons affect network properties of the DG-CA3 circuit thought to be important for pattern separation (Piatti et al., 2013). Sparseness of encoding is a well-characterized property of the DG that may support pattern separation by facilitating global remapping and ensuring that the same CA3 neurons are not activated by distinct ensembles of dentate granule neurons (O'Reilly and McClelland, 1994; Rolls, 1996). However, how adult hippocampal neurogenesis impacts sparseness of activation in DG or influences global remapping in the DG is not known. In theory, adult hippocampal neurogenesis may modulate sparseness of encoding in DG in several different ways. First, synaptic competition between dendritic spines of adult-born neurons and those of pre-existing mature granule neurons for entorhinal cortex (EC) inputs (Toni et al., 2007) may result in a redistribution of synaptic weights thereby affecting post-synaptic responses and consequently, sparseness of activation. Second, the addition of new mature neurons to the DG over time may facilitate input-expansion of entorhinal inputs and generation of sparser patterns of activation. Third, adult-born dentate granule neurons, may, in addition to functioning as cell-autonomous encoding units, also non-cell autonomously modulate the excitability of the mature dentate granule neurons (Sahay et al., 2011a; Kheirbek et al., 2012b). In this model, a small number of excitable young adult-born neurons exert feed-back inhibition onto the DG through their connections with hilar interneurons and mossy cells (Lacefield et al., 2010; Sahay et al., 2011a; Kheirbek et al., 2012b) and dictate sparseness of activation in DG and input specificity of mature neurons (Marin-Burgin et al., 2012). Together, increased sparseness of activation in DG and high input specificity of mature dentate granule neurons may support an input expansion model for decorrelation of entorhinal cortical inputs in DG to drive global remapping.

In this study, we set out to examine this third possibility and specifically, if selectively changing levels of adult hippocampal neurogenesis modifies excitability of DG and properties of local hilar-inhibitory circuitry. Past studies in mice have suggested that blockade of adult hippocampal neurogenesis results in increased activity in the DG using *in vivo* recordings of gamma bursts (Lacefield et al., 2010) or immediate early genes (Burghardt et al., 2012), but the mechanistic origins of these non-cell autonomous and network wide effects are unclear. Therefore, here we used VSDI in combination with laser photostimulation or electrical stimulation in the GCL, rather than entorhinal inputs, of *iBax^nestin^* mice and hippocampal x-irradiated mice that we had previously shown were better and impaired, respectively, in contextual fear discrimination learning (Sahay et al., 2011b), a task shown to require pattern separation (Niibori et al., 2012). We found reduced spread of neural activity and neuronal activation in the DG of *iBax^nestin^* mice, whereas blockade of hippocampal neurogenesis resulted in modest changes in VSD signal spread in the opposite direction. Since the VSD signal is generated throughout GCL, rather than just in the subgranular zone, it is likely to primarily reflect the activation of the mature dentate granule neuronal population.

**Figure 7:**
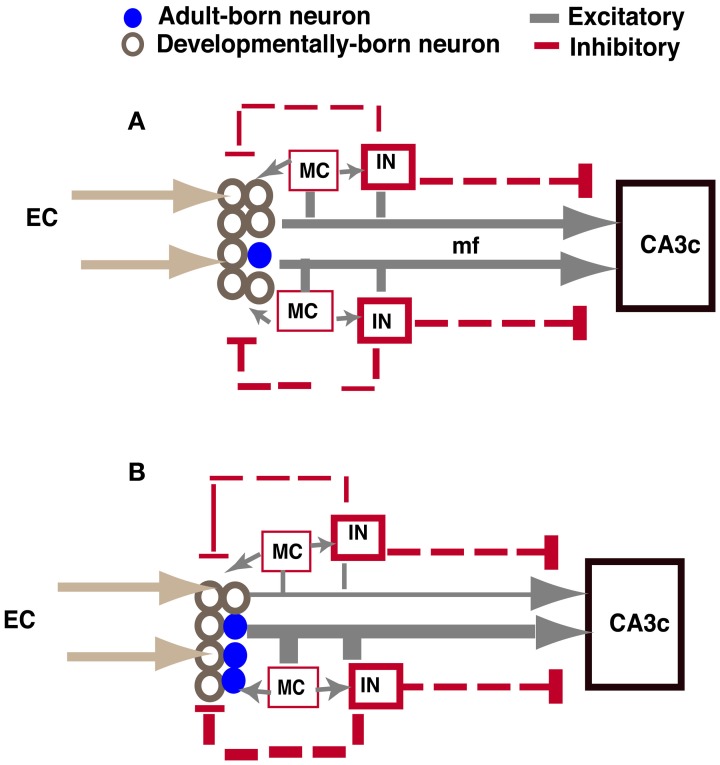
**Model showing how increasing adult hippocampal neurogenesis modulates DG excitability while maintaining output to CA3c (A).** In control animals, adult-born and mature dentate granule neurons exert feed-back inhibition onto mature dentate granule neurons through excitatory contacts with hilar interneurons. Young adult-born dentate granule neurons are insensitive to this feed-back inhibition. The extent of DG activation dictates the strength of feed-forward excitation and di-synaptic feed-forward inhibition to CA3c mediated by excitatory contacts of mature and adult-born dentate granule neurons onto CA3c neurons and CA3c neurons via SL interneurons, respectively. **(B)** When the number of adult-born dentate granule neurons is increased, excitatory drive onto hilar interneurons is increased, thereby increasing feed-back inhibition onto mature dentate granule neurons. Consequently, feed-forward excitation and di-synaptic feed forward inhibition to CA3c mediated by mature dentate granule neurons is decreased. However, feed-forward excitation and di-synaptic feed forward inhibition mediated by adult-born dentate granule neurons to CA3c is increased. Thus, the integration of young adult-born dentate granule neurons into the DG-CA3 circuit directly and indirectly dictates the extent to which feed forward excitation and inhibition is recruited by young adult-born and mature dentate granule neurons, respectively, to activate CA3c. IN: hilar or stratum lucidum interneuron, EC, entorhinal cortex; mf, mossy fiber; MC, mossy cell. Size of line indicates strength of connection.

As illustrated in Figure 7, increased excitatory drive mediated by adult-born neurons onto hilar interneurons and mossy cells in *iBax^nestin^* mice can lead to increased feedback inhibition onto the DG, which may account for the VSD changes observed. Analysis of functional connectivity of adult-born dentate granule neurons has revealed synaptic connections with hilar mossy cells and interneurons (Toni et al., 2008). Furthermore, mossy fibers have a disproportionately higher number of excitatory synaptic contacts onto hilar- and CA3-GABAergic interneurons than pyramidal neurons, and this property is reflected in the anatomical segregation of feed-forward inhibition and excitation in mossy fiber terminal filopodia or small MFTs and large MFTs, respectively (Acsady et al., 1998; Henze et al., 2000; McBain, 2008; Ruediger et al., 2011). Pharmacological (Zhang et al., 2010) or optogenetic modulation of hilar interneurons (Andrews-Zwilling et al., 2012) has been shown to influence inhibition onto dentate granule neurons. Since young adult-born neurons are insensitive to GABAergic inhibition, this neuronal population is unlikely to be affected by feed-back inhibition (Schmidt-Hieber et al., 2004; Ge et al., 2008; Marin-Burgin et al., 2012). Therefore, mossy cells and hilar interneurons are well-positioned to be recruited by adult-born dentate granule neurons to mediate feed-back inhibition onto mature dentate granule neurons and regulate sparseness of activation in DG and input specificity of mature dentate granule neurons (Buzsaki, 1984; Bragin et al., 1995; Scharfman, 1995; Freund and Buzsaki, 1996; Coulter and Carlson, 2007; Marin-Burgin et al., 2012; Scharfman and Myers, 2012; Yu et al., 2013). In accordance with these observations, genetic ablation of mossy cells results in increased excitability of the DG and impairs contextual fear discrimination learning (Jinde et al., 2012).

Based on this logic, we assessed changes in GABAergic inhibition onto the mature dentate granule neurons of *iBax^nestin^* mice. We examined both GABAergic innervation of the DG as well as pre- and postsynaptic properties of perisomatic inhibitory synapses of mature dentate granule neurons in the DG. Although the number and presynaptic properties of GABAergic inputs remained unchanged in the DG of *iBax^nestin^* mice, we observed a significant change in the distribution of inhibitory postsynaptic strength without changes in the average strength. Since our recording conditions and selection of mIPSCs were set to detect mainly proximal inputs (see Materials and Methods), this result suggests that some mature dentate granule neurons are readjusting the gain of their perisomatic inhibitory synapses. Since the average strength of inhibition was unchanged despite adjustment of individual synapses, it is likely that overall inhibitory tone is preserved in these mice. Therefore, we asked whether the number of excitatory synapses of adult-born neurons onto hilar neurons is increased in *iBax^nestin^* mice. We employed a retroviral based Cre recombination strategy to cell-autonomously visualize how connectivity of adult-born dentate granule neurons with hilar interneurons is affected following *Bax* recombination and whether, an increase in number of *Bax* negative adult-born dentate granule neurons (as seen in *iBax^nestin^* mice) also results in increased excitatory contacts with hilar interneurons. Using this approach we found that (i) unlike in CA3ab, small MFTs, rather than MFT filopodia, constitute the vast majority of excitatory pre-synaptic contacts with GAD67+ processes in the hilus, (ii) the average number of contacts of 4 weeks old adult-born dentate granule neurons with GAD67+ processes in hilus per small MFT did not change following *Bax* recombination, and (iii) that mice with 4 weeks old adult-born dentate granule neurons that underwent *Bax* recombination had significantly more small MFTs in the hilus relative to controls. Together, these observations suggest that an increase in survival of adult-born dentate granule neurons following Bax ablation in neural progenitors is accompanied by an increase in excitatory drive by adult-born neurons onto hilar interneurons. While this increased innervation of hilar interneurons by adult-born neurons may or may not explain the changes in distribution of perisomatic inhibition onto mature dentate granule neurons, it represents a plausible mechanism by which increasing adult hippocampal neurogenesis increases feedback inhibition onto the DG to enhance sparseness of activation. Consistent with this notion, a recent study found that blockade of adult hippocampal neurogenesis resulted in decreased feed-back inhibition onto dentate granule neurons, although these changes were only observed after a certain period of time (Singer et al., 2011).

Analysis of CA3c output in *iBax^nestin^* mice did not show a difference suggesting that the integration of adult-born dentate granule neurons into the DG-CA3 circuit does not affect CA3c output. Whether CA3ab properties are altered in these mice and following ablation of adult hippocampal neurogenesis remains to be addressed. Caveats notwithstanding, based on lack of an effect on CA3c excitability and changes observed in the DG of *iBax^nestin^* mice, we propose that adult-born dentate granule neurons dictate the balance between feedback inhibition onto the mature dentate granule neurons in the DG and feed forward inhibition mediated by stratum lucidum interneurons (SL) to govern CA3c activation (Figure 7). CA3c activation is dictated by di-synaptic feed forward inhibition arising from excitatory contacts of mature and adult-born dentate granule neurons onto SL interneurons and direct feed-forward excitation (McBain, 2008; Ruediger et al., 2011). This feed forward inhibition and feed-forward excitation onto CA3c is in turn modulated by the extent of DG activation. When the number of adult-born dentate granule neurons is increased, feed-back inhibition onto mature dentate granule neurons, but not young adult-born dentate granule neurons, is increased. Consequently, both feed-forward inhibition mediated by excitatory contacts of mature dentate granule neurons onto SL interneurons and direct feed-forward excitation by mature dentate granule neurons onto CA3c neurons is decreased. In contrast, feed-forward inhibition and excitation by adult-born dentate granule neurons onto CA3 neurons is increased. Thus, a trade-off in recruitment of feed-forward excitation and inhibition onto CA3c neurons maintains CA3c activation. In the intact brain, since hilar interneurons and mossy cells receive perforant path and neuromodulatory inputs (Leranth and Hajszan, 2007; Yu et al., 2013), the net effect of increasing adult neurogenesis on DG excitability may be bidirectionally influenced by the extent of activation of these afferent systems in a given context or behavioral state.

In conclusion, our studies demonstrate that increased integration of adult-born neurons decreases excitability of the DG that may facilitate sparseness of DG activation and suggest a role for hilar circuit-based mechanisms in this non-cell autonomous modulatory function of adult-born neurons in encoding. Future studies dissecting the causal recruitment of local connectivity by dentate granule neurons at distinct stages of maturation will inform how cell-autonomous and non-cell autonomous functions of adult-born dentate granule neurons govern pattern separation and completion operations in the DG-CA3 circuit.

## Author contributions

Amar Sahay conceived the study, Amar Sahay, Rene Hen, Hey-Kyoung Lee, and Xiangmin Xu designed research; Taruna Ikrar, Nannan Guo, Kaiwen He, Antoine Besnard, Sally Levinson, Alexis Hill, Hey-Kyoung Lee, Xiangmin Xu, and Amar Sahay performed research; Taruna Ikrar, Kaiwen He, Antoine Besnard, Sally Levinson, Hey-Kyoung Lee, Rene Hen, Xiangmin Xu, and Amar Sahay analyzed data; Amar Sahay and Xiangmin Xu wrote the paper.

### Conflict of interest statement

The authors declare that the research was conducted in the absence of any commercial or financial relationships that could be construed as a potential conflict of interest.
